# Prediction of Head Movement in 360-Degree Videos Using Attention Model

**DOI:** 10.3390/s21113678

**Published:** 2021-05-25

**Authors:** Dongwon Lee, Minji Choi, Joohyun Lee

**Affiliations:** 1 Department of Electrical and Electronic Engineering, Hanyang University, Ansan 15588, Korea; dw2689@hanyang.ac.kr; 2 Division of Electrical Engineering, Hanyang University, Ansan 15588, Korea; mjlilac97@hanyang.ac.kr

**Keywords:** LSTM, GRU, head movement, time-series prediction, machine learning, attention model

## Abstract

In this paper, we propose a prediction algorithm, the combination of Long Short-Term Memory (LSTM) and attention model, based on machine learning models to predict the vision coordinates when watching 360-degree videos in a Virtual Reality (VR) or Augmented Reality (AR) system. Predicting the vision coordinates while video streaming is important when the network condition is degraded. However, the traditional prediction models such as Moving Average (MA) and Autoregression Moving Average (ARMA) are linear so they cannot consider the nonlinear relationship. Therefore, machine learning models based on deep learning are recently used for nonlinear predictions. We use the Long Short-Term Memory (LSTM) and Gated Recurrent Unit (GRU) neural network methods, originated in Recurrent Neural Networks (RNN), and predict the head position in the 360-degree videos. Therefore, we adopt the attention model to LSTM to make more accurate results. We also compare the performance of the proposed model with the other machine learning models such as Multi-Layer Perceptron (MLP) and RNN using the root mean squared error (RMSE) of predicted and real coordinates. We demonstrate that our model can predict the vision coordinates more accurately than the other models in various videos.

## 1. Introduction

Virtual Reality (VR) is a simulated experience that is similar or different from the real world. VR can be applied to entertainment and education. Another type of VR is Augmented Reality (AR) which contains a combination of real and virtual worlds, real-time interaction, and accurate 3D registration of virtual and real objects [1]. To implement these systems, they require VR headsets to generate images, sounds, and other sensations. The headsets consist of a Head-Mounted Display (HMD) with a small screen in front of the eyes which shows 360-degree images.

Currently, VR systems are generally based on desktop computers containing a virtual world. In other words, it displays the virtual world on a regular desktop display without using any positional tracking equipment. However, as Sergio et al. presented an end-to-end system for VR and AR telepresence called Holoportation in 2016 [2], low-latency communication became a core issue. As it is not possible to guarantee the low-latency network in any place, we propose another method to implement a real-time VR and AR system. This method predicts the head movement of various users watching a 360-degree video with HMD. Therefore, it can automatically track the focus in the real-time VR system even though the network condition is poor.

Prediction is used in a variety of fields including economics and statistics. Recently, it is expanded to communication systems. The traditional prediction models are generally Moving Average, Autoregression, Autoregression Moving Average model [3]. These models have been used in economics or statistics based on correlations among the data. However, they are not suitable in communication systems because their data may have few correlations. Therefore, the novel prediction models are based on machine learning approaches such as Random Forest [4], Support Vector Machine [5], or neural network [6].

Neural networks have a variety of models and are generally classified into feedforward and Recurrent Neural Network (RNN). Feedforward neural network is the first and simplest type of artificial neural network. The information moves in only one direction, from the input to the output nodes. Therefore, there are no cycles or loops in the network. Single-layer perceptron and Multi-Layer Perceptron (MLP) are kinds of feedforward neural networks. On the other hand, in the RNN, nodes form a graph or cycle along a sequence. Due to this cycle, RNNs can deal with the internal state to process variable length of inputs [7].

Inspired by the neural networks, the attention model has appeared to solve two big problems of previous machine learning methods. First, as RNN tries to compress all the pieces of information in one fixed-size vector, information loss occurs. Second, there is a vanishing gradient problem, which is the chronic problem of RNN. In other words, when the input data are long, there is a phenomenon of poor quality. Therefore, in order to correct this phenomenon, an attention technique focuses on important data and deliver them directly to the decoder appeared.

In this paper, we propose a prediction algorithm using an LSTM or GRU model with an attention model. The contributions of our paper are as follows: (i) to our knowledge, it is the first work that predicts the head movement coordinates, which are kinds of time-series data, using attention technique, and (ii) we generate an attention model motivated by transfer learning [8] and online learning [9], and employ various experiments to verify the effectiveness of our algorithm.

We introduce related work regarding various models to predict time-series data and several methods to predict head movement in Section 2. Subsequently, we formulate the prediction problem and architecture and internal operations of the LSTM unit, GRU, and attention model [10] in Section 3. Then, we analyze a dataset that we are going to use and apply the algorithm to the head movement data, which is described in Section 4. Finally, we verify the result by playing the video and compare its performance with other prediction models in Section 5.

## 2. Related Work

### 2.1. Time-Series Data Prediction Models

Time-series data represent a series of data points listed in time order. There are various models for predicting time-series data. The models can be classified into linear and nonlinear models depending on the distribution of the data. Autoregressive integrated moving average (ARIMA) models [11] represent one of the linear models that has been widely used for time-series prediction. Adebiyi et al. [12] built the ARIMA model for stock price prediction and concluded that it has a strong ability for short-term prediction. In the 1990s, more various types of models using machine learning were introduced. Support vector machine (SVM) or support vector network is a linear model that analyzes data used for classification and regression. However, ARIMA and SVM models require parameters for prediction. Therefore, an additional algorithm is needed to obtain the optimal parameters since they determine the accuracy of the model. Xibin et al. [13] used the particle swarm optimization algorithm [14] to find the optimal parameters of the SVM model for predicting the real estate price.

However, setting the optimal parameters using these algorithms takes so much computation time, so there is a limitation of accurate prediction for these data using only the linear models. Artificial neural network (ANN) models can capture the nonlinear relationship in the data through a learning (or training) process. The ANN models can be generally divided into feedforward [15] and recurrent neural network (RNN) [16] depending on the structure of the network. Yi-Shian et al. [17] combined ARIMA and ANN model to improve the prediction performance. As a result, the combined model analyzes the linear part of the data with the ARIMA model and the nonlinear part with the ANN model. Shiblee et al. [18] created a multilayer perceptron (MLP) model, which is one type of feedforward neural network model, for predicting several types of time-series data such as Internet traffic, stock index, and petroleum sales data.

As the traditional RNNs have a vanishing gradient problem, long short-term memory (LSTM) and gated recurrent unit (GRU) models are mainly used as the representative RNN models. Sima et al. [19] proved that the LSTM model can perform better than the ARIMA model by experimenting on financial time-series data. PERCEIVE [20] used a 2-stage LSTM model to predict uplink throughput in cellular network. Yuxiu et al. [21] created an LSTM model with the random connection between nodes. Therefore, it reduced the total number of parameters to be trained and the computation load.

LSTM models are often combined with other deep learning models. LC-RNN [22] is a deep learning model with a combination of convolution neural network (CNN) and LSTM for traffic speed prediction. As the CNN model is suitable for analyzing images, it was used to capture the spatial traffic flow of a certain area. Then, the LSTM model estimated the time-series patterns from the extracted data. Guowen et al. [23] predicted short-term traffic flow with the GRU model. They performed time and spatial correlation analysis and extracted the traffic flow data as an input feature of the GRU model. Rui et al. [24] compared the performance of the LSTM and GRU model and concluded that both models did not show a big difference.

### 2.2. Head Movement Prediction Methods

There have been new challenges to 360-degree video processing. The resolution and bit rates of 360 videos are considerably higher than traditional two-dimensional videos. Therefore, a novel compression method is required to alleviate the network load while preserving the quality of experience for video streaming. One unique fact for viewing 360-degree videos is that the viewers only focus on the viewport, which is a small part of the whole 360-degree video. In other words, it is possible to apply the quality degradation outside of the viewport because this part is rarely seen by the viewers.

Based on this fact, Cornia et al. [25] proposed an Attentive Convolutional LSTM model that focuses on relevant location, which is usually called salience, in the image. Although this model predicts salience for two-dimensional images, it is a fundamental method to estimate salience for 360-degree videos. Zhu et al. [26] predicted a salient area for 360-degree images and created a scanpath that contains the variance of visual perception and attention. Stefano et al. [27] presented a trajectory-based viewport prediction algorithm by grouping past users exhibiting similar viewing trajectories using spectral clustering. They created a model of the viewport evolution overtime for certain groups. Afshin et al. [28] and Silvia et al. [29] also used clustering for the viewport prediction method that integrates viewport pattern information from the previous video frames.

However, the previous salience prediction method focuses on static scenes, so it is easy to generalize the eye fixation on a certain scene. However, on dynamic scenes, this method showed lower performance than the static scenes. Therefore, Yanyu et al. [30] explored gaze prediction in 360-degree videos. In other words, they predicted where a viewer will see in the future. Ching-Ling et al. [31] developed fixation prediction networks to predict the viewer fixation. HOP [32], the Historical viewport trajectory of viewers and Object tracking Prediction, is a deep learning-based viewport prediction model.

## 3. System Model

### 3.1. Prediction Problem

We consider a 360-degree video whose length is *T* time slots. *N* viewers have watched this video, and we record the time series of vision coordinate vectors of user *i* as $Y^{i}=\left\{y_{1}^{i},y_{2}^{i},\cdots ,y_{T}^{i}\right\}$ for i∈{1,⋯,N}, where $y_{t}^{i}$ is the vision coordinate vector at slot *t*. In the machine learning problem, the dataset is split into a training set and a test set. We choose the datasets of *M* people for the training set denoted as $X=\{Y^{1},Y^{2},\cdots ,Y^{M}\}$. Then, we select the test dataset from the rest of the viewers, $Y^{M+1},Y^{M+2},\cdots ,Y^{N}$. The goal of the prediction problem is to estimate $Y^{M+k}$, where k=1,2,⋯,N−M, using the previous data points. We use the set of time-series data to train a prediction model using machine learning, which will be explained in Section 3.2.

### 3.2. Sliding Window Method

For this prediction model, we use the sliding window method [33] when training a time-series dataset. It takes *w* previous data points as an input vector and computes one output data. In other words, $y_{j}^{i}=\left(y_{j-w+1}^{i},y_{j-w+2}^{i},\cdots ,y_{j}^{i}\right)$ is an input vector and ${\hat{y}}_{j+1}^{i}$ is an estimated value of $y_{j+1}^{i}$ using an internal function $\delta_{j}^{i}:y_{j}^{i}\to {\hat{y}}_{j+1}^{i}$, where *j* is an index such that w≤j≤T−1.

We can also adjust the time step of the output value, instead of estimating $y_{j+1}^{i}$. For example, if we want to estimate the output after *r* time steps, $\delta_{j}^{i}:y_{j}^{i}\to {\hat{y}}_{j+1}^{i}$ will be switched to $\delta_{j}^{i}:y_{j}^{i}\to {\hat{y}}_{j+r}^{i}$. The function $\delta_{j}^{i}$ will be approximated by a neural network introduced in Section 3.3. $\delta_{j}^{i}$ is updated for each time step until the function computes the final output value ${\hat{y}}_{T}^{i}$. To check that the function $\delta_{j}^{i}$ fits the training data, a loss function $L_{j}^{i}$ is defined. The function $L_{j}^{i}:\left(y_{j}^{i},{\hat{y}}_{j}^{i}\right)\to R$ computes the error between training and estimated data, e.g., $|y_{j}^{i}-{\hat{y}}_{j}^{i}|$, or a square of difference, ${\left(y_{j}^{i}-{\hat{y}}_{j}^{i}\right)}^{2}$.

After completing the learning process, the internal function $\delta_{T}^{M}$ computes *T* test samples ${\hat{Y}}^{M+k}=\left\{{\hat{y}}_{1}^{M+k},{\hat{y}}_{2}^{M+k},\cdots ,{\hat{y}}_{T}^{M+k}\right\}$ using the previous *w* data points, where ${\hat{y}}_{j}^{M+k}$ is the corresponding value of $y_{j}^{M+k}$ estimated by the function, where *j* is an integer from 1 to *T*. Then, we can evaluate the performance of the function by comparing elements in the real *T* test set $Y^{M+k}$ and the predicted set ${\hat{Y}}^{M+k}$. We compute the overall error by averaging the error of each data point, $|y_{j}^{M+k}-{\hat{y}}_{j}^{M+k}|$, or a square of difference, ${\left(y_{j}^{M+k}-{\hat{y}}_{j}^{M+k}\right)}^{2}$.

### 3.3. Methodology

**Figure 1:**
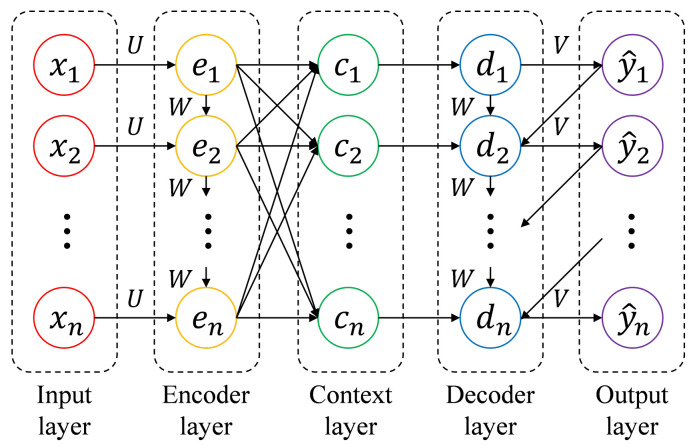
Structure of the Attention model.

The attention model [34] is an input technique for a neural network that focuses on certain features of input data. It is an improved model of the encoder–decoder model [35], which is designed to correspond to the various length of the input sequence. It allows the decoder to select information from the encoder by generating a different vector for every time step of the decoder and calculating it in the function of the previous hidden state and every hidden state of the encoder with weight *W*. Consequently, the Attention model adjusts importance to the various elements of the input sequence and focuses on more relevant inputs (Figure 1).

The encoder layer is a stack of recurrent units, such as RNN, LSTM, or GRU cells, which accept a single element of the input sequence $x_{t}$. Each hidden state $e_{t}$ is computed as an output of a function of weighted sum of the previous hidden state $e_{t-1}$ and the current input $x_{t}$. This process can be expressed as Equation (1).

The context vector $c_{t}$ is the output of the encoder layer and becomes the input for the decoder. It contains the information for the input sequence to allow the decoder to estimate the final output sequence. To calculate $c_{t}$, we compute the alignment score s(j,t), that is, a combination of *j*-th time step in the encoder and *t*-th time step in the decoder, expressed as Equation (2). In Equation (2), *W*, *U*, and *V* are weights of the model that are updated during the training process. *W* is the weight in the hidden states of the encoder, *U* is the weight in the input layer, and *V* is the weight in the hidden states of the decoder. The alignment score is normalized using softmax function expressed as Equation (3), and it is called the attention weight α(j,t). The attention weight determines the importance of the input of time step *j* for the output of time step *t*. Finally, the context vector is computed as the weighted sum of every hidden state of the encoder, expressed as Equation (4).

The decoder layer contains a stack of recurrent units, which accept $c_{t}$ as the input sequence of the decoder. The hidden state $d_{t}$ is computed as an output of a function of context vector $c_{t}$, the previous hidden state $d_{t-1}$, and the previous output ${\hat{y}}_{t-1}$. This process can be expressed as Equation (5). It enables to find the correlation between several input elements and corresponding output elements. Then, the final output is calculated by applying the softmax function to the weighted hidden state, expressed as Equation (6).

(1) $$e_{t}=f\left(We_{t-1}+Ux_{t}\right)$$

(2) $$s\left(j,t\right)=V\tanh\left(Ud_{t-1}+We_{j}\right)$$

(3) $$\alpha \left(j,t\right)=\frac{\exp s(j,t)}{\sum_{j=1}^{M}\exp s(j,t)}$$

(4) $$c_{t}=\sum_{j=1}^{T}\alpha \left(j,t\right)e_{j}$$

(5) $$d_{t}=f\left(d_{t-1},{\hat{y}}_{t-1},c_{t}\right)$$

(6) $${\hat{y}}_{t}=\frac{\exp Vd_{t}}{\sum_{t=1}^{n}\exp Vd_{t}}$$

## 4. Dataset and Model Description

### 4.1. Head Movement Dataset

In this section, we make a brief description of the dataset and analyze the data. The dataset used in this paper is a 360-degree video head movement dataset obtained from the navigation patterns of 59 users watching the videos with an HMD in 2017 [36]. The ages of users are from 6 to 62 with an average age of 34 years. Twenty percent of users are women and 61% of users have never used an HMD before. They watch five videos for ~70 s each.

The content of the videos is a diving scene, moving roller coaster, time-lapse of New York, virtual reconstruction of Venice, and guided tour of Paris, respectively. We name initials for each video and describe them in Table 1. Each video is available on YouTube searching for its YouTube ID. The spatial resolution of the videos is 3840 × 2048 pixels for all videos and the frame rate ranges from 25 to 60 fps (frame per second). Every 360-degree video is converted into an equirectangular format, which is one stitched image of 360 degrees horizontally and 180 degrees vertically. This dataset represents the head position using the unit Hamiltons quaternion, which is denoted as Equation (7):

(7) $$q=\left(q_{0},q_{1},q_{2},q_{3}\right)=\left(q_{0},q_{1}i+q_{2}j+q_{3}k\right)=\left(\cos\left(\theta /2\right),\sin\left(\theta /2\right)v\right)$$

where **i, j, k** are orthonormal bases, θ is a given angle, and **v** is an unit vector such that v=(x,y,z)=xi+yj+zk [37]. This quaternion expression has some advantages. It is simpler than the matrix representation, and it is not affected by the gimbal lock [38], which is a critical issue of the Euler angles representation [39]. The length of an epoch (time index) is the inverse of the frame per second (e.g., 33 ms for 30 fps).

Then, the dataset records the head positions for each frame using quaternion. Our main task is to predict these values using the machine learning models. In order to apply the data to the machine learning models, we normalize the value using min-max normalization, which sets the range of the data to [0, 1] given in Equation (8);

(8) $$y_{i}^{\prime }=\frac{y_{i}-\min\left(Y\right)}{\max(Y)-\min(Y)}$$

where $y_{i}$ is an original value, and $y_{i}^{\prime }$ is the normalized value. In learning, $y_{i}^{\prime }$ is used instead of $y_{i}$.

### 4.2. Correlation among Coordinate Components

We investigate correlation among four coordinate components, $q_{0},q_{1},q_{2},q_{3}$, in the head movement dataset. We use Pearson correlation coefficient [40] defined as Equation (9),

(9) $$r_{xy}=\frac{\sum_{i=1}^{n}\left(x_{i}-\bar{x}\right)\left(y_{i}-\bar{y}\right)}{\sqrt{\sum_{i=1}^{n}{\left(x_{i}-\bar{x}\right)}^{2}}\sqrt{\sum_{i=1}^{n}{\left(y_{i}-\bar{y}\right)}^{2}}}$$

where *n* is the number of data points, $x_{i}$ and $y_{i}$ are data points, and $\bar{x}$ and $\bar{y}$ are mean of data points, i.e. $\bar{x}=\frac{1}{n}\sum_{i=1}^{n}x_{i}$ and $\bar{y}=\frac{1}{n}\sum_{i=1}^{n}y_{i}$, in each component. $r_{xy}$ denotes the correlation coefficient of component *x* and *y*. The range of $r_{xy}$ is $|r_{xy}|\leq 1$, so if the absolute value of $r_{xy}$ is close to 1, then two components are highly correlated. Conversely, if $|r_{xy}|$ is close to 0, then two components are rarely correlated.

We choose several data samples in the head movement dataset and compute the correlation coefficients. The result of a data sample for the entire videos is shown in Table 2. We only denote indices of components for correlation. For example, $r_{13}$ denotes the correlation coefficient between $q_{1}$ and $q_{3}$.

If all components are correlated with each other, we can integrate coordinate components as one input vector. In other words, we can use $\left(q_{0},q_{1},q_{2},q_{3}\right)$ as an input vector of the model. As shown in Table 2, some components such as $q_{0}$ and $q_{2}$, $q_{0}$ and $q_{3}$, and $q_{1}$ and $q_{3}$ are correlated each other to a certain degree. However, other components have little correlation. We also discover that these correlations differ from each dataset. Therefore, we can use four coordinate components as one input vector of the prediction model when every component is correlated with each other. Otherwise, we must use each component individually as the model may learn incorrect correlations and degrades the performance.

### 4.3. Algorithm Description

The proposed attention model, generated by PyTorch machine learning library [41], is shown in Algorithm 1. As mentioned in Section 3.1, we combine *M* samples of *N* users’ datasets as one training set and select one dataset from the remaining samples as a test set. Then, we normalize the training set as shown in Equation (8). After processing the data with the min-max normalization, we decide the parameters of the attention model: numbers of input (*input*) and output (*output*) features, hidden layers in the encoder and decoder (*hidden*), and fully connected layers (*fc_layer*). The learning rate is *lr* and the number of data points in the training set is *n*. We also set the size of the sliding window *w* and time step *r* for the estimation. Then we train the model with these parameters.
**Algorithm 1** Prediction with an attention model.**Input:** time-series dataset $Y^{i}=\left\{y_{1}^{i},y_{2}^{i},\cdots ,y_{T}^{i}\right\}\left(i=1,2,\cdots ,M\right)$ for the training set and $Y^{M+k}=\left\{y_{1}^{M+k},y_{2}^{M+k},\cdots ,y_{T}^{M+k}\right\}\left(k=1,2,\cdots ,N-M\right)$ for the test set  1: Merge training samples into one training set $X=\left\{Y^{1},Y^{2},\cdots ,Y^{M}\right\}=\left\{y_{1},y_{2},\cdots ,y_{n}\right\}$  2: Normalize the training set using min-max normalization  3: Parameter: *input, output, hidden, f c_layer, lr, t, w,r*  4: Create an attention model with parameters *input, output, hidden, f c_layer*  5: **while** epoch ≤*t* **do**  6:  Compute an internal function $\delta_{i}:y_{i}=\left(y_{i-w+1},y_{i-w+2},\cdots ,y_{i}\right)\to {\hat{y}}_{i+r}$  7:  Apply adam optimization algorithm with initial learning rate lr  8:  Extract features from the hidden states in the encoder layer; $X\to \{e_{1},e_{2},\cdots ,e_{n}\}$  9:  Multiply the attention weight; $\left\{e_{1},e_{2},\cdots ,e_{n}\right\}\to \tilde{X}=\left\{c_{1},c_{2},\cdots ,c_{n}\right\}$  10:  Compute the output from the hidden states in the decoder layer; $\tilde{X}\to \left\{y_{1},y_{2},\cdots ,y_{n}\right\}$  11:  Compute the mean squared of loss function $L_{i}\;\left(i=1,\cdots ,n-r\right)$ such that $L_{i}=\frac{1}{n-r}\sum_{i=1}^{n-r}{\left({\hat{y}}_{i+r}-y_{i+r}\right)}^{2}$  12:  Apply back propagation and update the internal function; $\delta_{i}\to \delta_{i+1}$  13: **end while****Output:**${\hat{y}}_{j}^{M+k}\;\left(j=1,\cdots ,T\right)$, RMSE, and coefficient of determination (R2)

In the training process, we use the backpropagation method, which is one of the methods for computing the gradient in the multi-layer neural networks [42], and the adam optimization algorithm, which is one of the adaptive learning rate algorithms that can alternate the gradient while training [43]. As explained in Section 3.3, some meaningful features are extracted in the encoder layer from the training data. This process is expressed in Equation (10).

(10) $$e_{t}=v_{e}^{T}\tanh\left(W_{e}\left[h_{t-1};c_{t-1}\right]+U_{e}X_{t}\right),$$

where $e_{t}=\left\{e_{1},e_{2},\cdots ,e_{n}\right\}$ is an output vector from the encoder, $v_{e},W_{e},U_{e}$ are the parameters in the training, $c_{t-1}$ is the previous cell state of LSTM, and $h_{t}=f_{1}\left(h_{t-1},X_{t}\right)$ is the hidden state of the encoder with an input sequence $X_{t}=\left\{y_{t},y_{t+1},\cdots ,y_{t+w-1}\right\}$ from the input vector $X=\{y_{1},y_{2},\cdots ,y_{n}\}$ and a nonlinear function $f_{1}$ such as an LSTM unit.

Then, these features are multiplied with the attention weights and become the input of the decoder layer. Finally, we can get the output values from the hidden states in the decoder. The output vector can be denoted as Equation (11).

(11) $$d_{t}=v_{d}^{T}\tanh\left(W_{d}\left[g_{t-1};s_{t-1}\right]+U_{d}h_{t}\right),$$

where $v_{d},W_{d},U_{d}$ are the parameters in the training, $s_{t-1}$ is the previous cell state of LSTM, and $g_{t}$ is the hidden state of the decoder.

Applying these methods, we can get the loss function that can evaluate the training performance. This process is iterated for a certain number of times (epoch) and the value of the loss function got smaller as the model can estimate the values well in the training set.

When the training is completed, we can predict the data in the test set that has never been used for training with the trained model. To evaluate the performance of this model, we use root mean squared error (RMSE) and coefficient of determination (R2) defined as Equation (12) and square of $r_{\hat{y}y}$ in Equation (9), respectively, where ${\hat{y}}_{i}$ and $y_{i}$ indicate the predicted and real values, respectively.

(12) $$\mathrm{RMSE}=\sqrt{\frac{1}{n}\sum_{i=1}^{n}{\left({\hat{y}}_{i}-y_{i}\right)}^{2}}$$

## 5. Results and Evaluation

In this simulation, we first augment datasets of 40 people as one training set, then we use each dataset of the remaining 19 people as the test set. In the head movement prediction, the input and the output features are both the coordinate values. As the input and output features are stored in a one-dimensional array, the numbers of the input and output features are both 1. However, as mentioned in Section 4.2, if we can guarantee that all components are correlated with each other, we may use a coordinate vector $\left(q_{0},q_{1},q_{2},q_{3}\right)$ as an input of the model. In this case, we should set *input* as 4.

Then, we set *hidden* as 64 and *fc_layer* as 1, as too many hidden layers spend too much time for learning and may cause overfitting and fail to estimate the test data. We also set the time step for prediction considering the average length of time index in Table 1 and transmitting time to a video streaming server. For example, we set *w* as 4 and *r* as 100 for the “Diving” video, predicting the coordinate after 3 s. In this experiment, we set lr to 0.01 and iterate the training for 500 times. These parameter values are maintained the same in the following subsections unless specifically mentioned.

**Figure 2:**
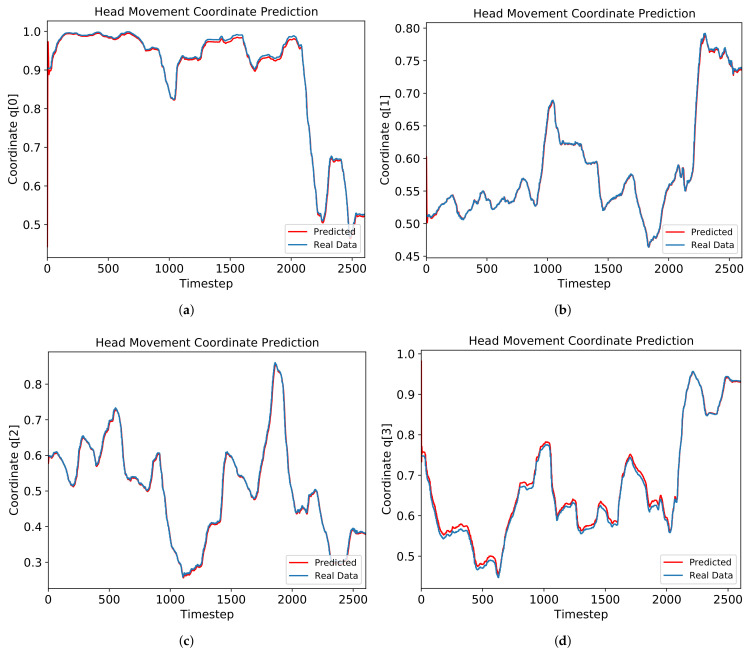
Prediction result for each quaternion component of head movement in the “Diving” video for coordinate (**a**) $q_{0}$, (**b**) $q_{1}$, (**c**) $q_{2}$, and (**d**) $q_{3}$.

**Figure 3:**
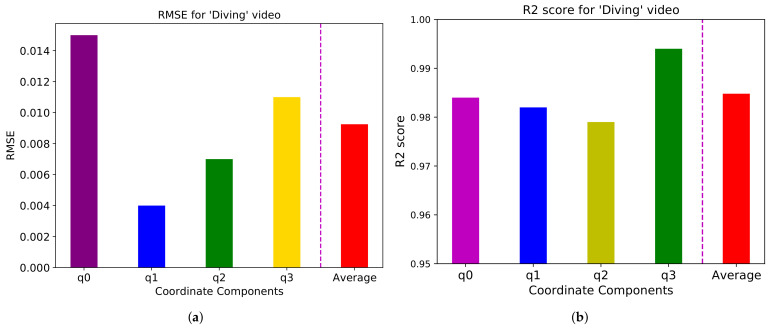
(**a**) RMSE and (**b**) R2 score for the “Diving” video for $q_{0}$, $q_{1}$, $q_{2}$, and $q_{3}$, and the average of four coordinates.

We conduct the simulation on a PC with Intel i7-9700KF CPU, NVIDIA GeForce RTX 2070 GPU, 64GB RAM, and Linux Ubuntu 20.04 operating system. The prediction result for the “Diving” video applying an attention model is shown in Figure 2. Each RMSE value for four quaternion components is shown in Figure 3a. The average RMSE for the “Diving” video is about 0.009, achieving approximately 90% prediction accuracy. R2 score for each component is shown in Figure 3b. The average R2 score for the “Diving” video is around 0.985.

We also measure the computing time for estimating the head movement coordinates of the video. In detail, we measure the average time it took to estimate the next data point in the test set and evaluate the performance of the model. As a result, the average computing time for training the model is around 300 microseconds on our PC.

In the following subsections, we compare the performance of our model with several criteria. We aim to prove that our model outperforms the previous models.

### 5.1. Machine Learning Models

**Figure 4:**
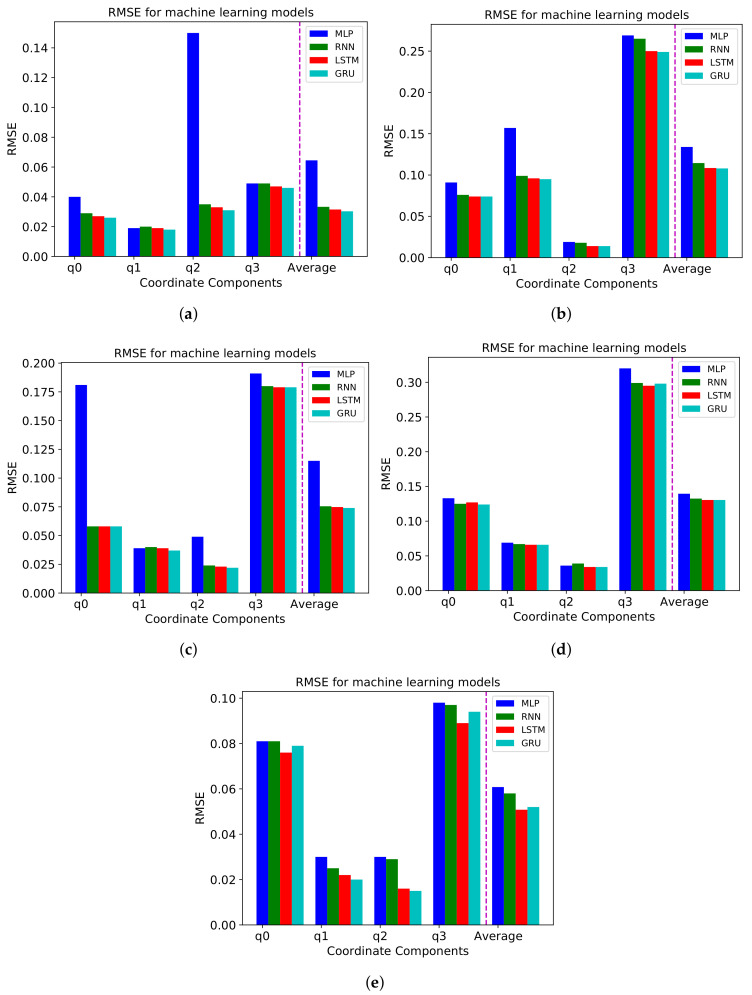
RMSE for MLP, RNN, LSTM, and GRU models for four coordinates and average (**a**) Diving, (**b**) Time-lapse, (**c**) Venice, (**d**) Rollercoaster, and (**e**) Paris video.

**Figure 5:**
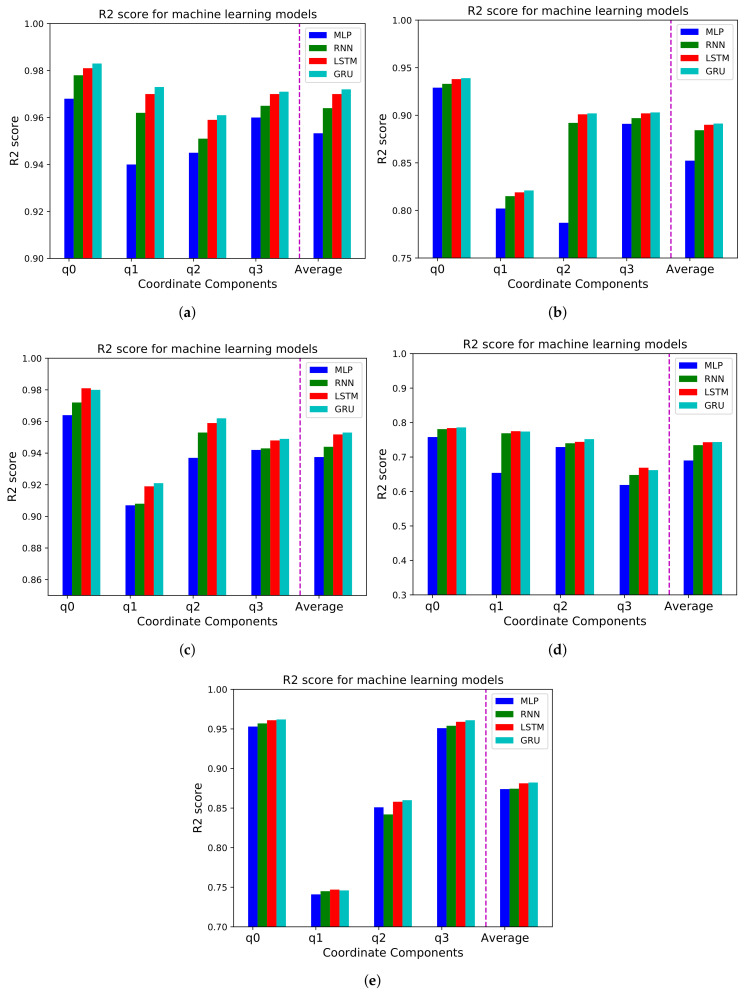
R2 score for MLP, RNN, LSTM, and GRU models for four coordinates and average (**a**) Diving, (**b**) Time-lapse, (**c**) Venice, (**d**) Rollercoaster, and (**e**) Paris video.

We compare the RMSE values and R2 scores for the entire videos applying MLP, RNN, and GRU model. We also compute an average of the RMSE and R2 values for all coordinate components. As shown in Figure 4 and Figure 5, we discover that the MLP model show the highest RMSE and the lowest R2 score, and LSTM and GRU models show the lowest value and the highest R2 score even though there is a little difference between LSTM and GRU model. Therefore, we can conclude that MLP model leads to the worst forecasting performance and LSTM and GRU model perform best among the four models.

### 5.2. Impact of Motions

**Figure 6:**
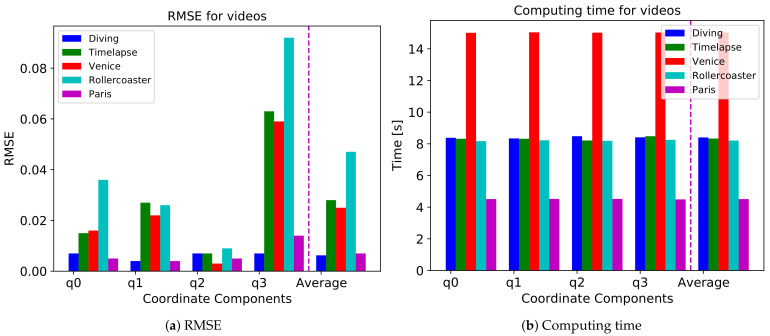
(**a**) RMSE and (**b**) computing time for ‘Diving’, ‘Timelapse’, ‘Venice’, ‘Rollercoaster’, and ‘Paris’ videos.

To see the impact of motions in the videos, we compare the performance with several videos. Some objects move slowly in the “Diving” and “Venice” videos, and there are many static objects in the “Paris” video. However, in the “Timelapse” and “Rollercoaster” videos, objects in the video move very fast and have a lot of motions. We compare the RMSE of each video with an LSTM model. The result is shown in Figure 6a including an average of four quaternion components. As a result, we find that the model shows better performance in the slowly moving videos than in the fast-moving videos.

We also compare the computing time for these videos. The result is shown in Figure 6b. The computing time is the longest for the “Venice” video and the shortest for the “Paris” video. Besides, the computing time is almost the same for the “Diving”, “Time-lapse”, and “Rollercoaster” videos. Therefore, we can conclude that the computing time is irrelevant to the motions of video and only relevant to the amount of the dataset, as the prediction model and its parameters are identical for every video.

**Figure 7:**
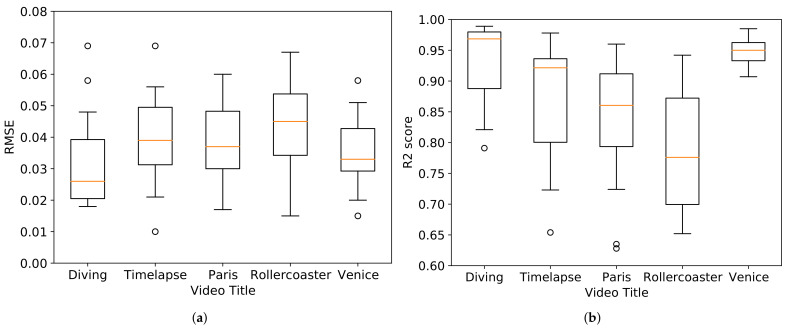
Box plot of (**a**) RMSE and (**b**) R2 score for each video.

We also conduct many simulations for various test sets by randomly selecting test sets, e.g., cross-validation. We randomly selected datasets for 40 people as a training set and tested the model for the remaining 19 people, respectively. Figure 7 shows the box plot of the RMSE and and R2 score for each video. For the “Diving” and “Venice” video, the RMSE and R2 score have a narrow range of minimum and maximum values with few outliers. As a result, we can conclude that the model has high generalization ability on these datasets. On the other hand, the RMSE and R2 scores have a wide range of minimum and maximum values for the “Paris” and “Rollercoaster” videos, which contain many fluctuations. Therefore, we can say that the model has low generalization performance on datasets with many fluctuations.

### 5.3. Impact of Attention

**Figure 8:**
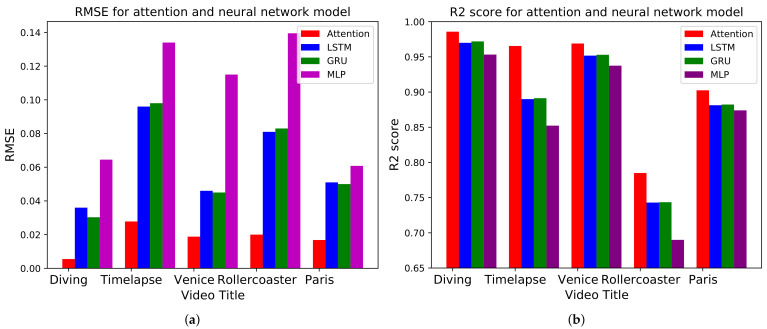
(**a**) RMSE and (**b**) R2 score for the attention and baseline LSTM, GRU, and MLP model without attention for ‘Diving’, ‘Timelapse’, ‘Venice’, ‘Rollercoaster’, and ‘Paris’ videos.

**Figure 9:**
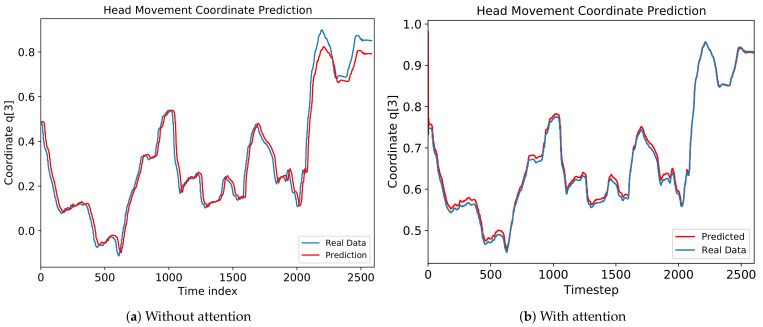
Comparison results (**a**) without and (**b**) with attention for coordinate $q_{3}$ in ‘Diving’ video.

As explained in Section 3.3, the attention model improves the performance of the prediction model. To see the importance of the attention model, we compare the performance of the attention and a baseline LSTM, GRU, and MLP model. Figure 8a,b show the comparison result with and without attention for every video. The RMSE is lower and the R2 score is higher in the attention model than the baseline models for all videos. Figure 9 shows the detailed comparison using the attention model. The prediction error is lower in Figure 9b than in Figure 9a, especially after 2000 time steps. As a result, we can conclude that applying the attention model reduces the prediction error and improves the performance of the fundamental neural network models for all videos.

### 5.4. Impact of Hyperparameters

There are many hyperparameters in machine learning. These hyperparameters are initialized to certain values when implementing the model. As mentioned in Algorithm 1, *hidden, lr, t, w, r* can be hyperparameters. In this subsection, we conduct experiments on various *hidden, lr*, and *t*. Experiments on time window (*w, r*) are implemented in Section 5.6. In these experiments, we use only ‘Diving’ video for prediction, as conducting on entire videos has been implemented in Section 5.2 thus it might be redundant. We adapt RMSE and R2 score for the performance evaluation metrics.

**Figure 10:**
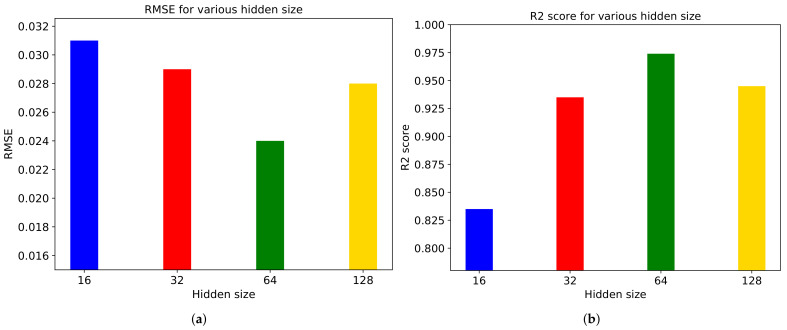
(**a**) RMSE and (**b**) R2 score for various hidden layers in ‘Diving’ video.

Figure 10 depicts performance metrics on various hidden layer size. We conduct experiments on 16, 32, 64, and 128 hidden layers. As a result, we can get the best performance on 64 hidden layers and the worst performance on 16 hidden layers. We conclude that too small number of hidden layers leads to the worst performance but too many hidden layers also degrade the performance of the model.

**Figure 11:**
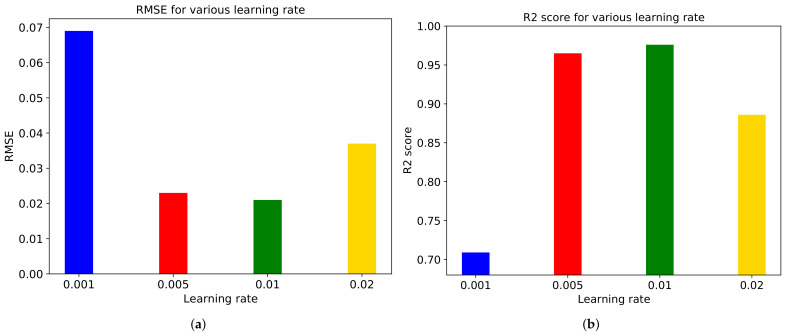
(**a**) RMSE and (**b**) R2 score for various initial learning rate in ‘Diving’ video.

We also conduct experiments on initial learning rates of 0.001, 0.005, 0.01, and 0.02, shown as Figure 11. We obtain the best performance at the rate of 0.01, which is the default setting of the model. Too low or high learning rate makes the model hard to converge, thus resulting in bad performance.

**Figure 12:**
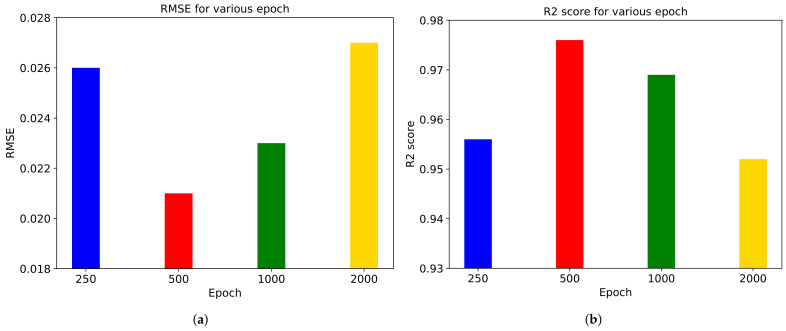
(**a**) RMSE and (**b**) R2 score for various epochs in ‘Diving’ video.

Figure 12 shows experimental results on various training epochs. The RMSE and R2 score are the best in 500 epochs, but we can say that more training epochs do not guarantee better performance. In other words, an overfitting occurs when the training epochs are too much.

### 5.5. Regularization

We apply several regularization methods in the LSTM model. The regularization methods used in the model are Dropout [44], AlphaDropout [45], and weight decay. In this subsection, we set the training epoch as 1000 and an initial learning rate of 0.02. We use the ‘Rollercoaster’ dataset for prediction and RMSE and R2 score for evaluation metrics.

**Figure 13:**
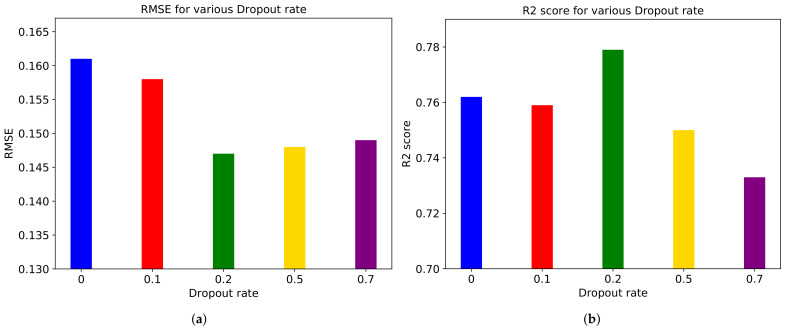
(**a**) RMSE and (**b**) R2 score for various Dropout rates in ‘Rollercoaster’ video.

Figure 13 shows performance metrics on various Dropout rates. The Dropout rate means the rate of not using neurons in the hidden layer. We use 0.1, 0.2, 0.5, and 0.7 as the Dropout rates. As a result, we found that the rate of 0.2 makes the optimal performance but too high Dropout rate degrades the performance of the LSTM model.

**Figure 14:**
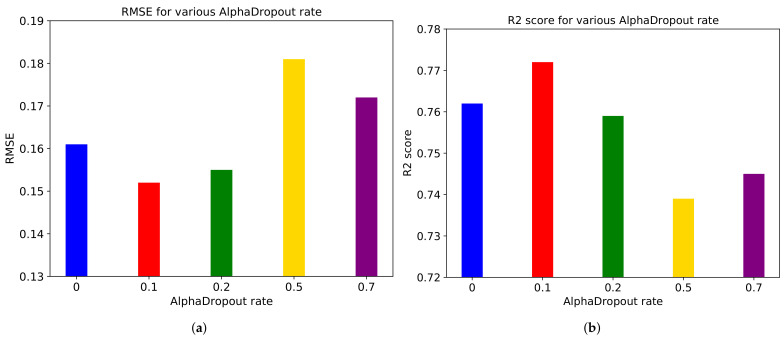
(**a**) RMSE and (**b**) R2 score for various AlphaDropout rates in ‘Rollercoaster’ video.

Figure 14 describes performance metrics on various AlphaDropout rates. We use the same rates as the Dropout rates. We conclude that the rate of 0.1 makes the optimal performance.

**Figure 15:**
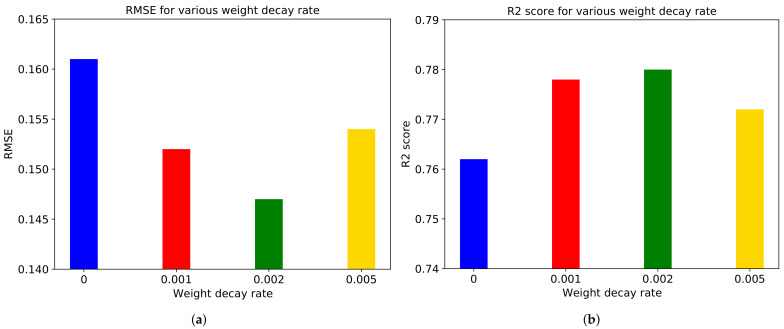
(**a**) RMSE and (**b**) R2 score for various weight decay rates in ‘Rollercoaster’ video.

We use the AdamW [46] optimization algorithm instead of Adam to see the impact of weight decay. AdamW adds weight decay in Adam. We choose 0.001, 0.002, and 0.005 as rates of weight decay. From the experimental result depicted in Figure 15, we can say that the weight decay rate of 0.002 leads to the best performance.

These regularization methods show similar performance under each optimal rate. Even though the regularization generally improves the performance the model without regularization, excessive Dropout or AlphaDropout rate degrades the performance. These methods discard some nodes in the input and hidden layer, thus removing too much nodes might result in bad performance.

### 5.6. Time Window

**Figure 16:**
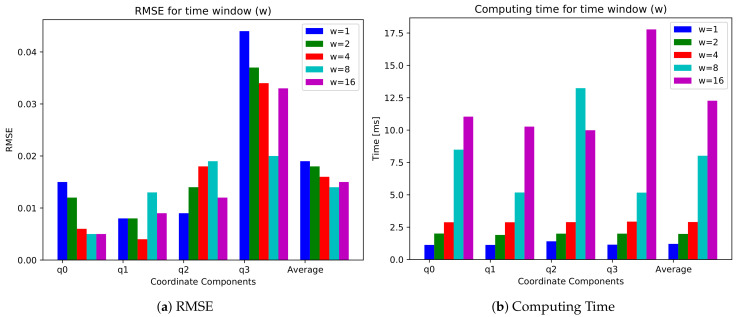
(**a**) RMSE and (**b**) computing time for window sizes of 1, 2, 4, 8, and 16.

In time-series data prediction, a prediction model uses past data in the previous time steps to estimate the future data. This method is called the sliding window or window for short. Specifically, an input data with window size of *w* can be denoted as $\left\{y_{i-w+1},\cdots ,y_{i-1},y_{i}\right\}$. We set the window size of 4 as a default value and vary the window size. We compute the RMSE of each coordinate for ‘Timelapse’ video with different window sizes. We also measure the computing time to estimate the results. As shown in Figure 16, the smaller window size reduces the computing time but increases the RMSE. In contrast, the larger window size decreases the RMSE but the computation time is sharply increased.

### 5.7. Comparison with Other Models

**Figure 17:**
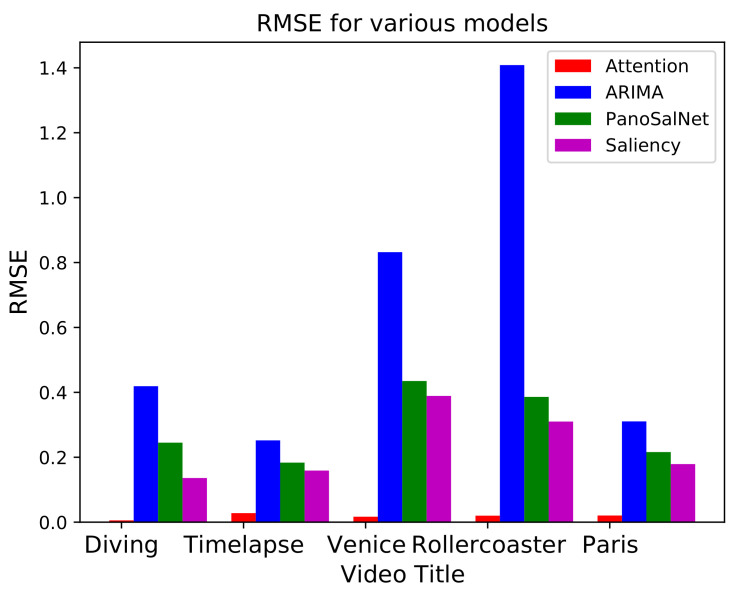
RMSE for attention, ARIMA, PanoSalNet, and Saliency model.

In this subsection, we compare the RMSE values with other models in the previous studies. We choose PanoSalNet [47], Saliency [25], and ARIMA models for comparison. The ARIMA model requires three parameters; p,d, and *q*. *p* is the order of autoregression, *d* is the order of differencing, and *q* is the order of the moving average [48]. In this simulation, we set parameters (p,d,q) as (1, 10, 0) for comparison. However, these parameters can be altered to other values such as (2, 10, 0) or (1, 15, 0) for certain datasets due to LU decomposition error [49]. The result is shown in Figure 17. The ARIMA model performs the worst prediction accuracy. In addition, it can only accept univariate data, i.e., we cannot use $\left(q_{0},q_{1},q_{2},q_{3}\right)$ as an input vector of the ARIMA model. Therefore, we can conclude that the ARIMA model is unsuitable for the head movement prediction.

### 5.8. Displaying the Results

**Figure 18:**
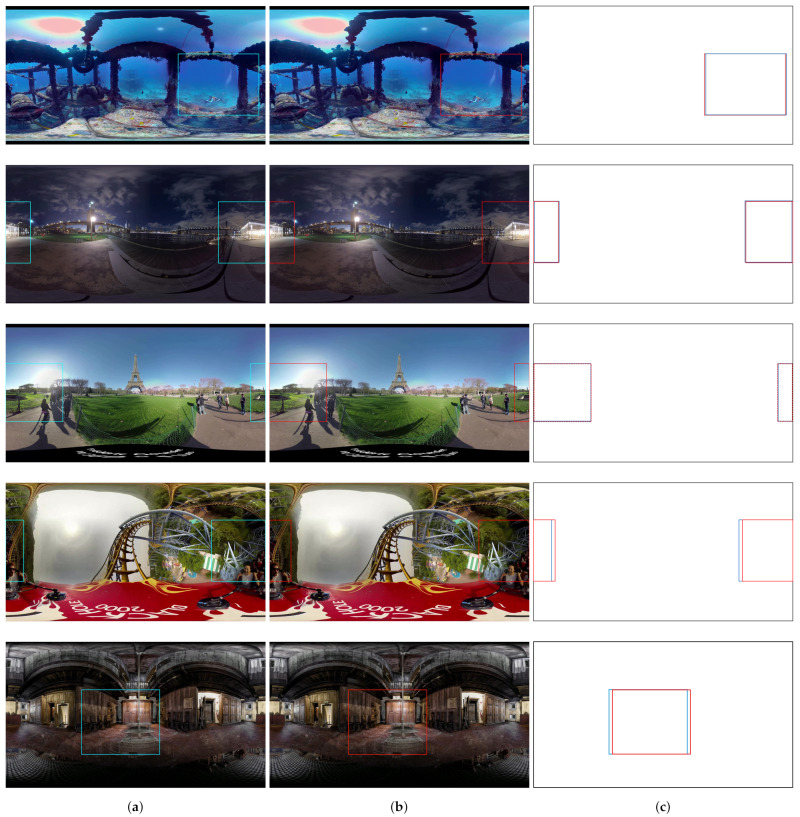
Prediction result for partial video frames (**a**) original movement, (**b**) predicted movement, and (**c**) overlapped frames of head movement.

In order to verify that these predicted values work well in the video, we apply an algorithm that can display the head movement into a rectangular area. Figure 18a,b show some captured frames of the video indicating the head movement. The blue rectangle represents the movement of the original dataset, and the red rectangle represents the movement of the predicted dataset. Figure 18c shows the overlapped frames of the original and predicted video. As shown in Figure 18, the area of the predicted movement almost covers the area of the original movement. Therefore, we can conclude that the attention model predicts head movement well.

## 6. Conclusions

In this paper, we created a prediction model based on the Attention model, which is one of the machine learning methods using RNN. Furthermore, in order to evaluate the performance of the model, we used RMSE to numerically validate the accuracy of the model and the algorithm to represent the head movement into a rectangular area. Then, we compared the performance with the other types of machine learning to verify that the proposed model can obtain the best accuracy. The simulation results also show that the Attention model can guarantee the highest performance compared with fundamental machine learning models.

Although we have proposed the prediction model, there are some limitations. This model is supposed to run under certain conditions. As the neural networks require training data, a segment of the file should be equipped in advance. In other words, the model cannot predict the entire data without the training data. In addition, it may take a considerable time to train the data depending on the parameters of the model and the specification of the device. If the training time is longer than the playback time of the video, uninterrupted video streaming will be impossible.

## Figures and Tables

**Table 1:** Description of the videos used for head movement prediction.

Name	YouTube ID	Content Description	Frame Rate [fps]	Average Length of Time Index [ms]
Diving	2OzlksZBTiA	Diving scene	30	33
Timelapse	CIw8R8thnm8	Timelapse of streets in New York	30	33
Paris	sJxiPiAaB4k	Virtual guided tour of Eiffel Tower district	60	16
Rollercoaster	8lsB-P8nGSM	Riding a rollercoaster	30	33
Venice	s-AJRFQuAtE	Virtual reconstruction of Venice	25	40

**Table 2:** Pearson Correlation Coefficient for head movement dataset.

Correlation	$r_{01}$	$r_{02}$	$r_{03}$	$r_{12}$	$r_{13}$	$r_{23}$
Diving	0.2	−0.2	0.5	0.01	0.5	−0.2
Timelapse	−0.07	−0.1	0.5	0.2	0.3	−0.08
Paris	0.004	−0.2	0.6	0.2	0.2	−0.1
Rollercoaster	0.1	−0.7	0.6	0.01	0.6	−0.4
Venice	0.05	−0.1	0.5	0.1	0.3	−0.06

## Data Availability

The datasets in this paper can be obtained from the following link: https://dl.acm.org/do/10.1145/3193701/full/, accessed on 23 March 2020.

## Abbreviations

The following abbreviations are used in this manuscript:

VR	Virtual Reality
AR	Augmented Reality
HMD	Head-Mounted Display
MA	Moving Average
ARMA	Autoregressive Moving Average
ARIMA	Autoregressive Integrated Moving Average
ANN	Artificial Neural Network
SVM	Support Vector Machine
CNN	Convolution Neural Network
MLP	Multi-Layer Perceptron
LSTM	Long Short-Term Memory
RNN	Recurrent Neural Network
GRU	Gated Recurrent Unit
RMSE	Root Mean Squared Error
